# Supplementary material for: TREM2 Expression and Amyloid-Beta Phagocytosis in Alzheimer’s Disease
Source: Int J Mol Sci. 2023 May 11;24(10):8626. doi: 10.3390/ijms24108626 (PMC10218378; doi:10.3390/ijms24108626)
Supplement: Supplementary file 1 [file ijms-24-08626-s001.zip › ijms-2346535-supplementary.pdf]

Figure S1

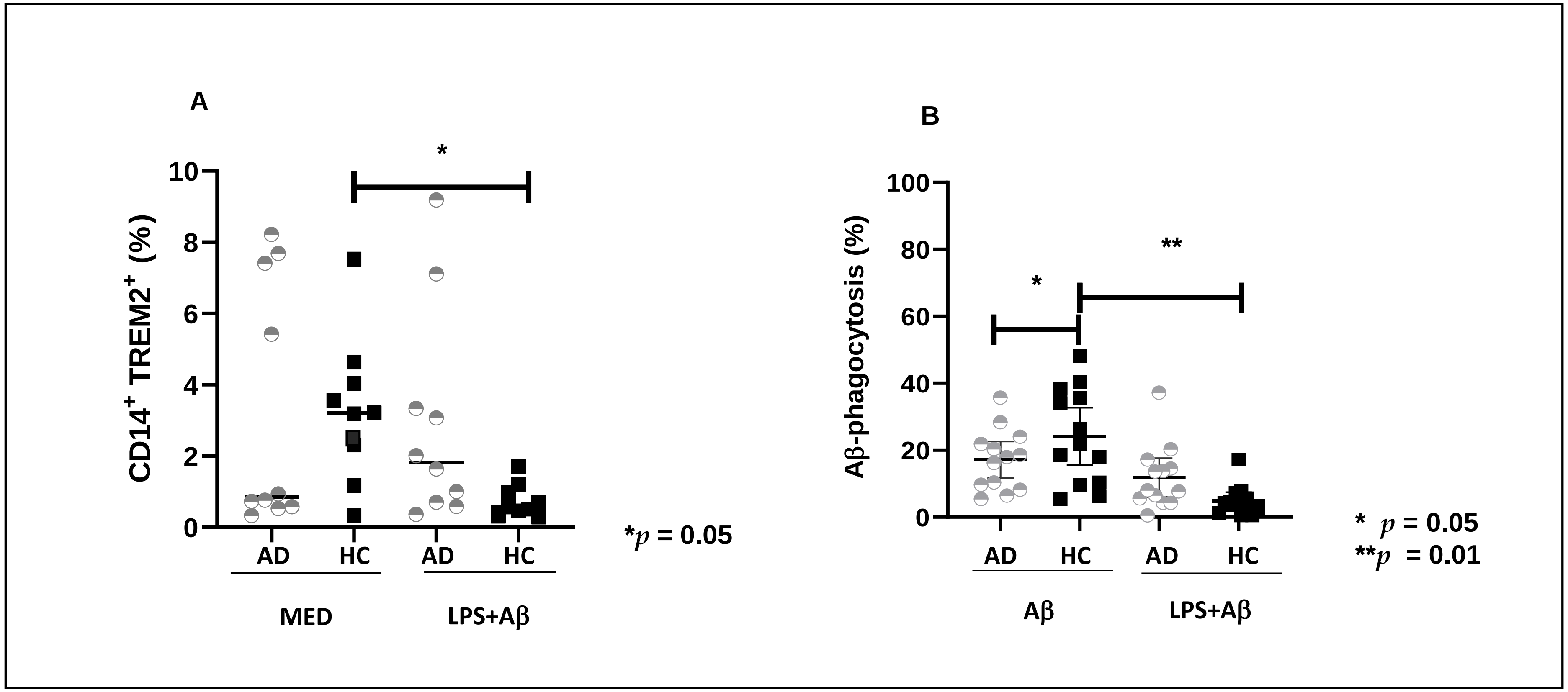

Figure S1: (A): TREM2-expressing CD14⁺ monocytes in 10 Alzheimer's Disease patients (AD) and 10 healthy controls (HC); cells were cultured in medium alone (MED) or upon LPS and Aβ stimulation. The Mann Whitney test was used to compare the percentage of TREM2-expressing CD14⁺ monocyte in AD and HC, and Wilcoxon matched-pair test for comparison before and after LPS stimulation. *p* values of less than 0.05 were considered significant (* $p = 0.05$). (B): Aβ42-FAM-phagocytosis by Aβ- or Aβ+ LPS- stimulated monocyte of 13 Alzheimer's Disease patients (AD) and 12 healthy controls (HC). Results are expressed as the percentage of monocytes phagocyting Aβ42-FAM. Analysis was performed by FlowSight. Mann Whitney test was used to compare the phagocytosis percentage in AD and HC and Wilcoxon matched-pair test for comparison before and after LPS stimulus. Horizontal lines represent median. *p* values of less than 0.05 were considered significant (* $p = 0.05$), (** $p = 0.01$).

Figure S2

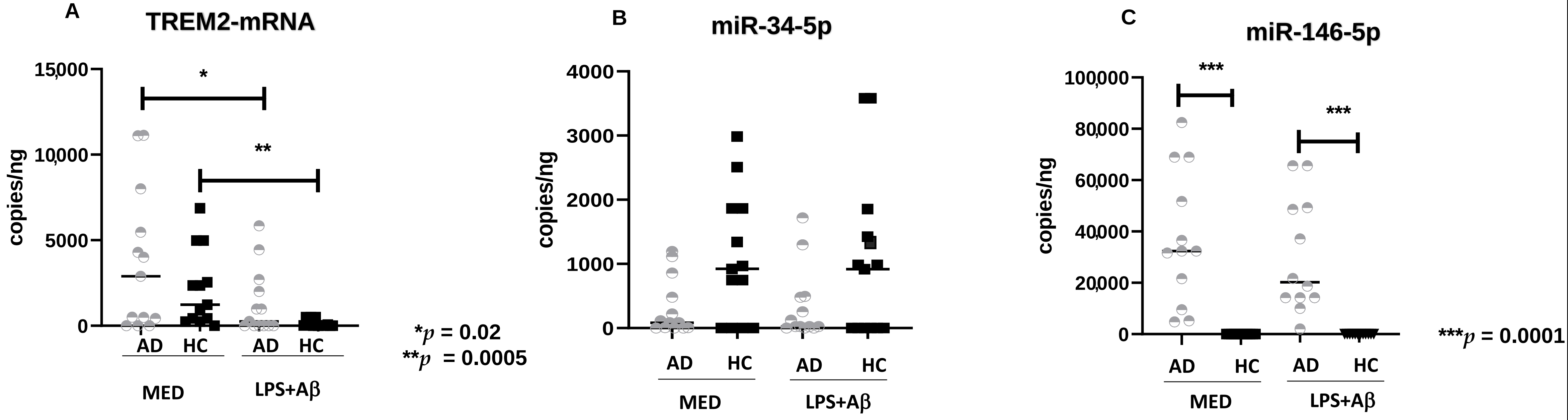

Figure S2: (A): Expression of TREM2 mRNA in unstimulated (MED) or LPS + Aβ_{42} stimulated- PBMC of 13 Alzheimer's Disease patients (AD) and 12 healthy controls (HC). (B): miR-34a-5p and (C): miR-146a-5p expression in unstimulated (MED) or LPS + Aβ_{42} stimulated-PBMC from 12 Alzheimer's Disease patients (AD) and 12 healthy controls (HC). The Mann Whitney test was used to compare the mRNA or miRNA expression; the Wilcoxon matched-pair test was used to compare results obtained in different cultural conditions. For ddPCR analysis, the QuantaSoft software version 1.7.4.0917 (Bio-Rad, Hercules, CA, USA) was used to quantify mRNA and miRNAs copies. Horizontal lines represent median. *p* values of less than 0.05 were considered significant: * $p = 0.02$; ** $p = 0.005$; *** $p = 0.0001$).

Figure S3

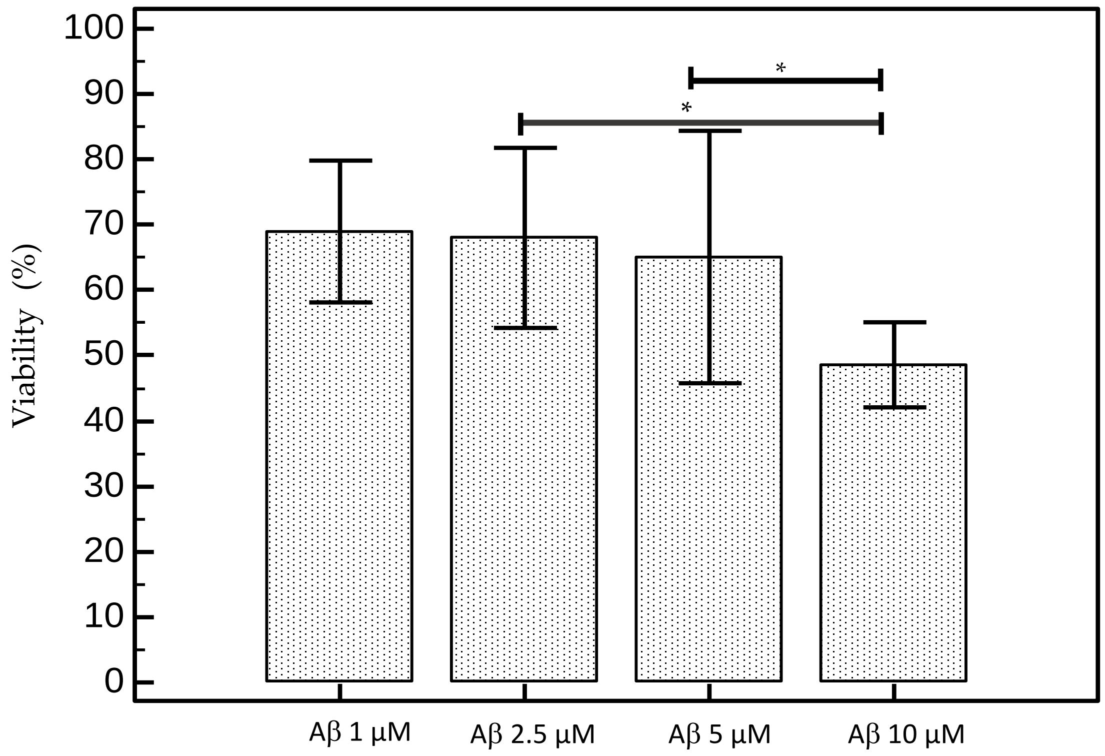

Figure S3: Results of MTT (3-(4,5-dimethylthiazol-2-yl)-2,5-diphenyltetrazolium bromide) assay of Amyloid-beta (Aβ) on monocytes–derived to PBMC from Alzheimer's disease patients and controls enrolled in the study. For cell viability, cells were cultured in a 96-wells plate with serial Aβ concentrations (1 to 10uM)Viable cells with active metabolism convert MTT into a purple colore. Adsorbance was read at 570 nm using a plate reading spectrophotometer. Values are expressed as mean ± standard deviation of the mean (SD); *p* values of less than 0.05 were considered significant (* $p = 0.002$).
